# Recent developments of tools for genome and metabolome studies in basidiomycete fungi and their application to natural product research

**DOI:** 10.1242/bio.056010

**Published:** 2020-12-02

**Authors:** Fabrizio Alberti, Saraa Kaleem, Jack A. Weaver

**Affiliations:** 1 School of Life Sciences and Department of Chemistry, University of Warwick, Gibbet Hill Road, Coventry CV4 7AL, UK; 2 School of Life Sciences, University of Warwick, Gibbet Hill Road, Coventry CV4 7AL, UK

**Keywords:** Basidiomycete fungi, Mushrooms, Natural products, Secondary metabolites, CRISPR/Cas9

## Abstract

Basidiomycota are a large and diverse phylum of fungi. They can make bioactive metabolites that are used or have inspired the synthesis of antibiotics and agrochemicals. Terpenoids are the most abundant class of natural products encountered in this taxon. Other natural product classes have been described, including polyketides, peptides, and indole alkaloids. The discovery and study of natural products made by basidiomycete fungi has so far been hampered by several factors, which include their slow growth and complex genome architecture. Recent developments of tools for genome and metabolome studies are allowing researchers to more easily tackle the secondary metabolome of basidiomycete fungi. Inexpensive long-read whole-genome sequencing enables the assembly of high-quality genomes, improving the scaffold upon which natural product gene clusters can be predicted. CRISPR/Cas9-based engineering of basidiomycete fungi has been described and will have an important role in linking natural products to their genetic determinants. Platforms for the heterologous expression of basidiomycete genes and gene clusters have been developed, enabling natural product biosynthesis studies. Molecular network analyses and publicly available natural product databases facilitate data dereplication and natural product characterisation. These technological advances combined are prompting a revived interest in natural product discovery from basidiomycete fungi.

This article has an associated Future Leader to Watch interview with the first author of the paper.

## Abstract (precis)

**Summary:** Fungi of the phylum Basidiomycota can make bioactive natural products. We discuss recent advances of tools for genome and metabolome studies that will facilitate natural product discovery and characterisation.

## Introduction

**Fig. 1.**
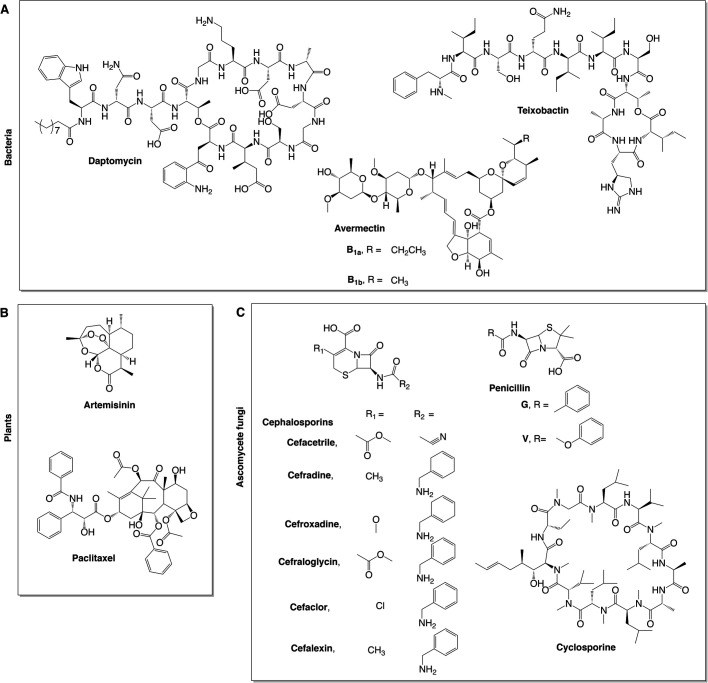
**Structures of NPs mentioned in this article produced by organisms other than basidiomycete fungi.** (A) Bacterial, (B) plant, and (C) ascomycete NPs.

Natural products (NPs) are low-molecular weight molecules that are made by living organisms and are often referred to as secondary metabolites. This is because, despite offering a competitive advantage to the producing organism, they are not essential for its survival. NPs are used by humankind for many applications, including as drugs; NPs and NP derivatives represent 25% of new anticancer and 55% of new antibacterial drugs approved by the US Food and Drug Administration (FDA) between 1981 and 2019 (Newman and Cragg, 2020). Historically, selected groups of organisms have been the main focus for NP discovery; one such group of organisms is actinomycete bacteria, from which a variety of bioactive NPs (Fig. 1A) have been employed in modern human and veterinary medicine, and agriculture. These NPs include the antibiotic daptomycin, isolated from *Streptomyces roseosporus* (Debono et al., 1988); and anthelmintic agents avermectins, derived from *Streptomyces avermitilis* (Miller et al., 1979). Gram-negative bacteria are another important reservoir for NPs of varying bioactivities, including antibiotics, such as teixobactin (Fig. 1A), found in the recently discovered *Eleftheria terrae* (Ling et al., 2015). Plants represent another large source of NPs (Fig. 1B), including the antimalarial artemisinin, found in *Artemisia annua* (Klayman et al., 1984); and anticancer agent paclitaxel, marketed as Taxol, discovered in *Taxus brevifolia* (Rowinsky and Donehower, 1995).

Fungi are also a rich source of NPs, with NP discovery primarily focusing on species belonging to the phylum Ascomycota thus far. Various NPs from ascomycete fungi hold prominent roles in our daily lives (Fig. 1C), including β-lactam antimicrobials such as penicillin (Fleming, 1929) and cephalosporins (Abraham et al., 1953), originally discovered in *Penicillium rubens* and *Acremonium strictum*, respectively; and immunosuppressant cyclosporine, made by *Tolypocladium inflatum* (Kahan, 1989). Fungi of the phylum Basidiomycota represent a rich source of NPs, often overlooked for several key reasons, such as their large and complex genome structures, as well as being difficult to work with, as a consequence of their slow growth rate compared with other fungi and bacteria. Due to this, secondary metabolite study has lagged behind in basidiomycete fungi. With the release of an increasing number of whole-genome sequences (at present, more than 540 basidiomycete genomes are accessible from the web portal of the US Department of Energy Joint Genome Institute), as well as their antiquity in traditional medicine and culinary usage, the interest in the study of NPs in basidiomycete fungi has grown considerably in recent years.

As commonly seen in other microorganisms, and to some extent in plants, the genes that code for fungal NP biosynthetic enzymes are often clustered together on the same genomic region, a feature that allows researchers to more easily mine the genetic determinants of NP biosynthesis (Osbourn, 2010). Biosynthetic gene clusters can include genes coding for synthase enzymes, tailoring enzymes and transcriptional regulators (Medema et al., 2015).

## Basidiomycete fungi and their NPs

Fungi belonging to the phylum Basidiomycota can produce NPs that belong to several different biosynthetic classes (Lin et al., 2019), including terpenoids, polyketides, non-ribosomal peptides, ribosomally synthesised and post-translationally modified peptides, and indole alkaloids. Each class of NPs are structurally diverse and encompass their own biosynthetic enzymatic machinery and tailoring enzymes. Interclass differences may also be vast, with individual metabolites varying in structure, bioactivity, and size.

### Terpenoids

**Fig. 2.**
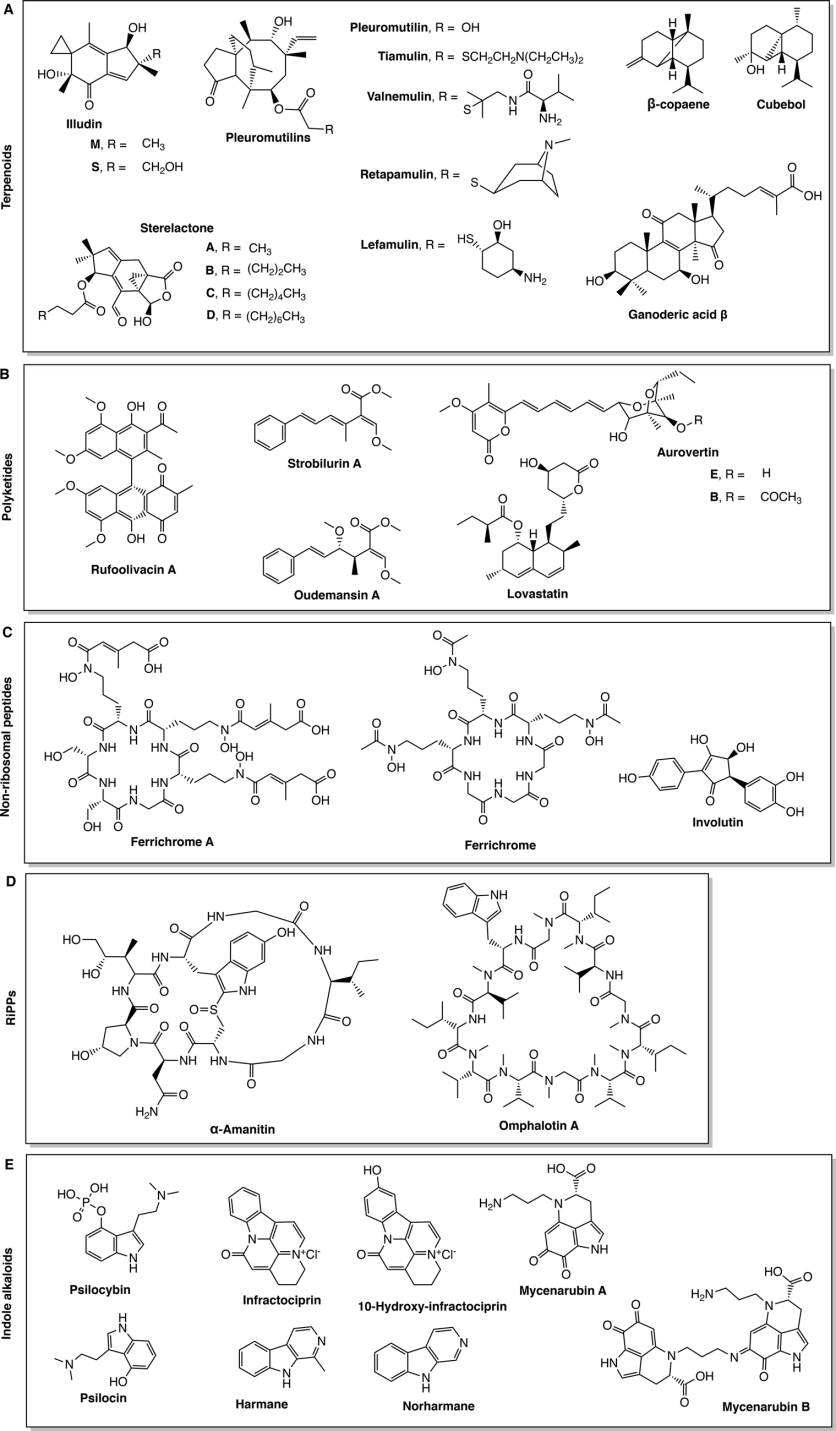
**Structures of NPs mentioned in this article produced by basidiomycete fungi.** (A) Terpenoids, (B) polyketides, (C) non-ribosomal peptides, (D) RiPPs, (E) indole alkaloids.

Terpenoids (Fig. 2A) are the most abundant and diverse class of NPs produced by basidiomycete fungi (Schmidt-Dannert, 2015), and are found in many other organisms, including plant and bacterial species. These NPs have a broad spectrum of bioactivities; some examples of terpenoids include: ganoderic acid β, one of several analogues of ganoderic acid made by *Ganoderma lucidum*, with this particular analogue possessing antiviral bioactivity (Min et al., 1998); illudin-S and illudin-M are two sesquiterpenoids with antitumour and antibiotic bioactivity, and are believed to be responsible for the toxicity of the jack-o'-lantern mushroom *Omphalotus illudens* (McMorris and Anchel, 1965); and antifungal sterelactones isolated from a *Stereum* sp. (Opatz et al., 2008). A particularly important example of a bioactive terpenoid made by basidiomycete fungi is that of the antibiotic pleuromutilin, a diterpene antibacterial NP, initially discovered in the two fungi *Pleurotus mutilus* (synonymous to *Clitopilus scyphoides* f. *mutilus*) and *Pleurotus passeckerianus* (synonymous to *Clitopilus passeckerianus*) during the golden age of antibiotic discovery (Kavanagh et al., 1949). Pleuromutilin mediates its antibacterial bioactivity by inhibition of protein synthesis through selective binding at the peptidyl transferase centre, at the A- and P-sites of the ribosome (Davidovich et al., 2007). Since its discovery, semi-synthetic derivatives tiamulin and valnemulin have been employed in veterinary use, and retapamulin is utilised as a topical antibiotic in humans (Daum et al., 2007; Poulsen et al., 2001). More recently, lefamulin has been granted permission by the US FDA for use in the treatment of community-acquired bacterial pneumonia, becoming the first ever antibiotic derived from basidiomycete fungi to be approved for systemic use in humans (Watkins and File, 2020).

Terpenoids all consist of several isoprene units, however, diversification in structure can be achieved by differences in linearity or cyclicity, saturated or unsaturated bonds, and other modifications, such as reduction, decarboxylation, glycosylation, alkylation, and acetylation, to name a few. For details on the biosynthesis of fungal terpenoids we refer the reader to other reviews on this topic (Hoffmeister and Keller, 2007; Schmidt-Dannert, 2015).

### Polyketides (PKs)

PKs are known to be highly abundant in fungi, with research primarily focusing on ascomycete fungi thus far. Despite this, known PKs of basidiomycete fungi are structurally and functionally diverse (Fig. 2B). Some examples of PKs produced by fungi of the phylum Basidiomycota include the antioxidant anthraquinone-related pigments, such as rufoolivacins, isolated from *Cortinarius purpurascens* (Bai et al., 2013; Gao et al., 2010); oudemansin, an antifungal antibiotic isolated from *Oudemansiella mucida* (Anke et al., 1979); mycotoxins aurovertin B and E from *Albatrellus confluens* (Wang et al., 2005); and lovastatin, an HMG-CoA reductase inhibitor used as a cholesterol lowering agent and found in the oyster mushroom, *Pleurotus ostreatus* (Atli and Yamac, 2012). Strobilurin A is an antifungal PK, first isolated from *Strobilurus tenacellus* (Steglich and Schramm, 1977). Strobilurin A mediates a fungicidal effect by selective targeting of the β-methoxyacrylate toxophore to complex III of the mitochondrial electron transport chain, inhibiting adenosine triphosphate synthesis (Becker et al., 1981). Since its discovery, derivatives of strobilurin A based on the β-methoxyacrylate moiety have been developed for use as agricultural fungicides, including the highest-selling fungicide azoxystrobin (Bartlett et al., 2002; Sauter et al., 1999). Strobilurin fungicides are estimated to have a market value of $3.4 billion, which corresponds to 25% of the overall global market of fungicides (Nofiani et al., 2018).

Most fungal PKs are synthesised by iterative multidomain type I polyketide synthase (PKS) enzymes, which are similar to fatty acid synthases (FASs), however some examples of type III PKSs are also known, which include a single ketosynthase domain (Seshime et al., 2005). For an overview of the biosynthetic mechanism of fungal PKs we refer the reader to other reviews on this topic (Cox, 2007; Hoffmeister and Keller, 2007).

### Non-ribosomal peptides (NRPs)

NRPs are a class of NPs largely underrepresented in basidiomycete studies (Fig. 2C), with research on these secondary metabolites being primarily focused on ascomycete fungi. Some NRPs found in fungi of the phylum Basidiomycota include ferrichrome and ferrichrome A, siderophores produced by *Ustilago maydis* (Winterberg et al., 2010; Yuan et al., 2001); and involutin, an Fe3+ reductant isolated from the ectomycorrhizal *Paxillus involutus* (Shah et al., 2015).

Fungal NRPs are synthesised by multimodular NRP synthetase (NRPS) enzymes, using either proteinogenic or non-proteinogenic amino acids to construct the NP (Vassaux et al., 2019).

### Ribosomally synthesised and post-translationally modified peptides (RiPPs)

Peptide NPs can be synthesised by two mechanisms. One is by the previously mentioned NRPSs, and another is ribosomally-driven synthesis. Unlike NRP synthesis, ribosomal biosynthesis requires the translation of a precursor peptide which must then be post-translationally modified, yielding the mature peptide. Fungal RiPPs (Fig. 2D) are relatively newly discovered, with the first family described in 2007 (Hallen et al., 2007). α-amanitin, isolated from *Amanita bisporigera*, is a ribosomally-synthesised amatoxin with a bicyclic octapeptide structure, and is the lethal constituent of this poisonous mushroom, exerting a toxic effect by selective inhibition of RNA polymerase II and consequently halting mRNA synthesis (Chafin et al., 1995). Omphalotin A is a potent nematicidal cyclic dodecapeptide isolated from the jack-o'-lantern mushroom *O. olearius* (Mayer et al., 1997). Until recently, omphalotin A was considered to be a product of NRPSs, however it has since been shown that incorporation of α-*N*-methylation of the peptide backbone occurs via the RiPP pathway (Ramm et al., 2017; van der Velden et al., 2017).

Commonly between RiPPs and other NPs is that their biosynthetic pathways are encoded by gene clusters. For an overview of RiPP biosynthesis in fungi and common post-translational modifications, we refer the reader to another recent review (Vogt and Künzler, 2019).

### Indole alkaloids

Indole alkaloids are one of the largest classes of nitrogen-containing secondary metabolites and are found across all kingdoms of life, however, fungi, particularly ascomycetes, are reportedly prolific producers of these NPs. Indole alkaloids are also well known in basidiomycete fungi (Fig. 2E), for example norharmane and harmane, tremorgenic neurotoxins isolated from the ivory waxy cap *Hygrophorus eburneus* (Teichert et al., 2008); dimeric pyrroloquinolines mycenarubin A and mycenarubin B are red pigments isolated from *Mycena rosea* (Peters and Spiteller, 2007); and acetylcholinesterase inhibitors infractopicrin and 10-hydroxyinfractopicrin isolated from *Cortinarius infractus* (Geissler et al., 2010). *Psilocybe* mushrooms have long been known for their use in both medicinal and religious practices (Andersson et al., 2008). Psilocybin and psilocin, the hallucinogenic alkaloids, were first identified in *Psilocybe* mushrooms in 1958, and shown to have profound psychotropic effects, similar to those induced by lysergic acid diethylamide (Hofmann et al., 1958). Psilocybin is a prodrug that rapidly dephosphorylates, forming the psychoactive, hallucinogenic psilocin. In humans, this dephosphorylation occurs in the liver, likely by first-pass metabolism, allowing psilocin to act as a high-affinity partial agonist for 5-HT_2A_ receptors in the central nervous system.

Indole alkaloids are generally derived from tryptophan, which is derived from the shikimic acid pathway, and dimethylallyl pyrophosphate, from the mevalonate pathway whose products also act as the precursor units for terpenoids. Though tryptophan is commonly used as an amino acid precursor, amino acids other than tryptophan, such as tryptamine, are known to be used in indole alkaloid biosynthesis (Xu et al., 2014).

## Recent advances in tools for the study of basidiomycete NPs

### Long-read whole-genome sequencing

Fungi belonging to the phylum Basidiomycota have an average genome size of 46 Mb (Mohanta and Bae, 2015). As a comparison, fungi of the phylum Ascomycota have an average genome size of 37 Mb (Mohanta and Bae, 2015), whereas the genomes of Archaea and Bacteria normally fall within 6 Mb (Kellner et al., 2018). This means that while bacterial genomes can be sequenced using short-read sequencing, accurate genome assembly in fungi requires long-read sequencing. This can be achieved in different ways, such as through the inexpensive nanopore sequencing (Dreamer et al., 2016). Indeed, nanopore whole-genome sequencing of basidiomycete isolates has been reported; for instance, the 54 Mb-genome of the plant pathogen *Rhizoctonia solani* was successfully sequenced through MinION and assembled into 606 contigs with an N50 of 199 Kb (Datema et al., 2016 preprint). More recently, MinION was used to identify all taxa present in a mock fungal community, including basidiomycete isolates, indicating that it may be employed to profile fungal communities (Mafune et al., 2019).

Despite the increased read length and reduced cost of nanopore long-read sequencing, the accuracy of base calling – 85–94% depending on the sequencing method (Jain et al., 2017) – and lower throughput than the established short-read platforms such as Illumina are an issue. However, when nanopore long-read sequencing is utilised in conjunction with short-read sequencing it is possible to assemble genomes of higher quality than either can provide independently. The quality of genomes assembled using a hybrid of long and short reads will only improve as the software for assembling these genomes also advances, for example Pilon now contains a function that will incorporate both Illumina and nanopore sequence data when polishing assemblies; while these software packages often focus on bacterial genomes, there is no doubt robust programs focussing on fungal genomes will soon follow (Goldstein et al., 2019), and multiple high-quality fungal genomes have already been sequenced using one flow cell in a MinION (McKenzie et al., 2020). Most *de novo* genome assembly programs today still utilise de Bruijn graph-based methods. This method is inappropriate for the quantity of indels and error rates produced by long-read sequencers today, therefore a return to the overlap-consensus method will likely improve the quality of assemblies made using long-read sequencing data. This has already been proven for bacteria, where an *Escherichia coli* genome purely assembled from nanopore sequencing data was used to create a single contig genome with 99.5% base accuracy using the ‘nanocorrect’ and ‘nanopolish’ pipeline (Loman et al., 2015). Furthermore long-read whole-genome sequencing techniques can detect base modifications, with the nanopore able to recognise all five C5-cytosine variants (Wescoe et al., 2014). It can detect endogenous and exogenous RNA modifications (Stephenson et al., 2020 preprint). Long-read sequencing is also unparalleled for examining metagenomic data, with recent studies assembling over 1000 high quality genomes from metagenomic samples run on the PromethION (Singleton et al., 2020 preprint). As nanopore technology matures we will likely see the accuracy of reads increase to be as competitive as short-read sequencing, recent advances have utilised neural networks within the base calling software to reach 97.7% accuracy (Silvestre-Ryan and Holmes, 2020 preprint), and further improvements to the hardware and software are likely in the coming years. It should also be noted that in the future, nanopore technology will not just be capable of long-read sequencing RNA and DNA, but will also be capable of sequencing proteins (Ouldali et al., 2020). Long-read sequencing of proteins will no doubt prove invaluable to groups searching for the biosynthetic pathways responsible for producing NPs.

### CRISPR/Cas9-mediated engineering of basidiomycete fungi

It has long been known that genetically transforming targets is a vital tool for detailed molecular analysis, and until recently it was exceptionally difficult to do this in basidiomycete fungi. Gene disruption, for example, has until recently, only been achieved using homologous recombination with a low efficiency (Ohm et al., 2010). However, protocols utilising CRISPR/Cas9 have now been developed for selected basidiomycete fungi (Table 1).

**Table 1.**
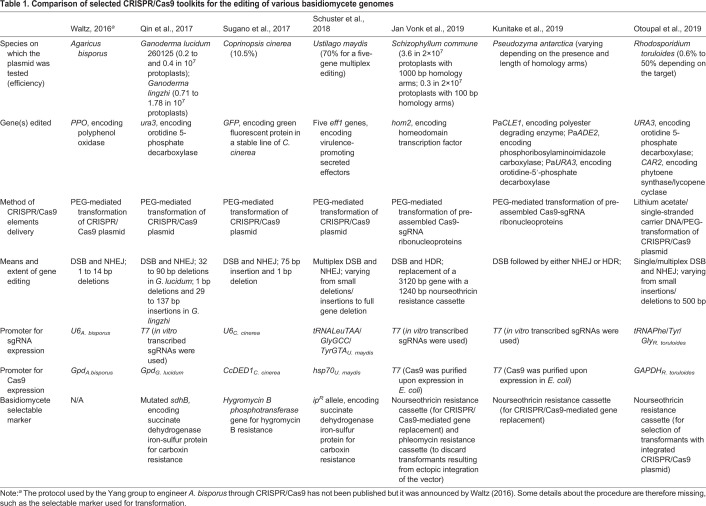
**Comparison of selected CRISPR/Cas9 toolkits for the editing of various basidiomycete genomes**

However, basidiomycete utilisation of CRISPR/Cas9 is more complex than working with bacteria, as Cas9 must be both codon-optimised and have a nuclear localisation signal attached. CRISPR/Cas9 systems have a multitude of advantages over other techniques, for example, target sites need only have a PAM sequence, which makes almost every gene a valid target. Compared with techniques such as transcription activator-like effector nucleases (TALEN) and zinc finger nucleases (ZFN), CRISPR/Cas9 is faster, more precise and versatile (Deng et al., 2017). Furthermore, it is viable for multiple simultaneous gene edits, making a dearth of selection markers less problematic. Most importantly, is the simplicity of components required, synthetic guide (sg)RNA and Cas9 being the major parts.

The breakthrough of CRISPR/Cas9 in basidiomycete fungi happened in 2016, when it was announced that Yinong Yang and collaborators used this technique to remove one of the genes that encodes polyphenol oxidase in *Agaricus bisporus*, slowing down the process of browning and increasing the shelf life of the button mushroom (Waltz, 2016). Despite the achievement being covered in news articles, the CRISPR/Cas9 method used by the Yang group to engineer *A. bisporus* has not been published. The following year a CRISPR/Cas9-based editing protocol was published for *G. lucidum* and *Ganoderma lingzhi* (Qin et al., 2017). The authors achieved disruption of the *ura3* gene in these fungi through CRISPR/Cas9-guided double stranded break, followed by repair through non-homologous end joining (NHEJ), which resulted in short insertions and deletions at the target sites. In the same year CRISPR/Cas9 was used to form the basis of a high-throughput transformation technique, which successfully inserted luciferase and GFP into *Coprinopsis cinerea* to assess constitutive promoters; the authors also developed an improved way of cryopreserving protoplasts (Sugano et al., 2017). Despite these successes, targeting efficiency remained low, potentially due to inefficient sgRNA and/or *Cas9* expression (Sugano et al., 2017); however, the gates of CRISPR/Cas9 for engineering and editing fungi of the phylum Basidiomycota were opened. A potential solution to this bottleneck has been provided in other fungi by utilising tRNA promoters to drive transcription of sgRNA. This was successfully performed on the ascomycete fungus *Aspergillus niger*, and where the Cas9 and sgRNA were on the same plasmid, this created gene mutations with an efficiency of ∼97% (Song et al., 2018). Once CRISPR/Cas9 was shown as a viable choice for the engineering of basidiomycete fungi it did not take long for a multitude of fresh techniques and improvements which expand the scope and increase the efficiency of CRISPR/Cas9 to be developed. For example, *Schizophyllum commune* has been successfully used to demonstrate pre-assembled Cas9-sgRNA ribonucleoproteins for use in gene deletion; the sgRNA, Cas9, and a repair template, were all inserted into protoplasts using PEG-mediated transformation, removing the need to optimise sgRNA and Cas9 expression within the host (Jan Vonk et al., 2019). Recombination events were selected for by the inclusion of a selectable marker cassette within the repair template. This technique is best described as CRISPR/Cas9 mediated homology-directed repair (HDR): a technique in which a double stranded break is repaired by providing a homologous DNA fragment. It can be used for the precise disruption of genes and insertion of selectable markers or other sequences of interest. The authors showed that homology arms of 250 bp were sufficient to efficiently induce the target gene replacement with a selectable marker cassette. Similarly, protoplasts of the basidiomycete yeast *Pseudozyma antarctica* have been efficiently transformed and had targeted genes disrupted via incubation with a ribonucleoprotein complex, consisting of the Cas9 protein and a single sgRNA with donor DNA containing a selectable marker (Kunitake et al., 2019). The authors showed that increasing the volume of the ribonucleoprotein mix increased transformation efficiencies, and that having homology arms of over 100 bp was best practice for inducing efficient homologous recombination with this system, although homology arms were shown to not actually be required to insert the donor DNA into the target locus. *Ustilago* *maydis* is another model organism that has recently seen successful CRISPR/Cas9-based multiplex gene editing, in which five genes coding for virulence-promoting secreted effectors were successfully inactivated using a single CRISPR plasmid (Schuster et al., 2018). Multiplexing is an important function for gene disruption in fungal pathways as usually biosynthetic routes are controlled by multiple genes and it is desirable to generate simultaneous knockouts. The capability of CRISPR/Cas9 systems to perform multiple simultaneous gene knockouts is where great potential lies. The previous techniques utilising ZFN and TALEN are laborious, and limited in scope in comparison, and we are seeing more multiplexing being performed in fungi of the phylum Basidiomycota now that the technology is maturing. For example, *Rhodosporidium toruloides* has been successfully multiplexed with CRISPR/Cas9 to disrupt the *car2* and *ura3* genes in a single transformation, and single transformations reached up to 50% efficiencies (Otoupal et al., 2019). Additional CRISPR/Cas9 platforms for successfully editing the NP-producer *G. lucidum* have recently been established (Liu et al., 2020; Wang et al., 2020a). Interestingly, it has been shown that by introducing an intron upstream of the *cas9* gene, it is possible to drastically increase efficiency of transformations by over ten times the rates seen without the intron, arriving at a final gene deletion rate of 36.7% (Liu et al., 2020). The inclusion of an intron before the start of the coding sequence is an established approach that has been shown to be necessary to achieve heterologous gene expression in several basidiomycete fungi, such as *C. cinerea*, *A. bisporus* (Burns et al., 2005) and *Armillaria mellea* (Ford et al., 2016).

As it stands, the problem of CRISPR in basidiomycete fungi is not just a case of low efficiency rates and lack of platforms and support, which are being overcome as shown above, but of off-target effects, screening and selection issues, continued genome modification after the error gene is repaired, and NHEJ indel size variation leading to frameshifts that cannot be controlled. Techniques have been developed to inhibit native NHEJ by using small molecules, like Scr7, allowing the HDR presented in templates we insert to dominate (Maruyama et al., 2015). Techniques such as this will continue to be developed and will eventually cross over not just into fungi but specifically into basidiomycete strains. A new method called PEM-seq has been developed that has high sensitivity and uses high-throughput sequencing; it can identify off-target sites of Cas9 and quantify the cutting efficiency of CRISPR/Cas9 at the target site, therefore, it can be used to find more efficient Cas9 cutting sites (Yin et al., 2019). This technique is not established in fungi of the phylum Basidiomycota yet, but it or technology like it are likely to arrive. As CRISPR/Cas9 matures we will see more and more basidiomycete-based platforms for high-throughput, rapid genome engineering. And no doubt as improvements and novel uses for CRISPR/Cas9 are discovered in other organisms and particularly other fungi, these changes too will eventually make their way to basidiomycete-based application. For example, a plasmid containing the *Aspergillus nidulan*s AMA1 sequence (Gems et al., 1991) has been generated for use in *Aspergillus* *oryzae*, which improves efficiency by being multi-copy, causing an increase in Cas9 and sgRNA, but a quirk of the AMA1-based plasmids is that they are discarded from the cells after several rounds of non-selective subculturing, theoretically making it possible to have infinite rounds of genetic engineering (Katayama et al., 2019). This will help to not just study biosynthetic pathways of NPs but also to generate fungal strains that have industrial application.

### Platforms for heterologous expression of basidiomycete gene cluster

While working on the NP producing basidiomycete itself is a powerful tool, once putative biosynthetic genes have been identified, few techniques are as powerful as insertion of these genes into heterologous hosts. Furthermore, while the NPs of basidiomycete fungi may be of medical and industrial interest, the fungi themselves are often not appropriate for generating industrial quantities of the NP due to their slow growth, furthering the need for robust platforms for heterologous expression of biosynthetic pathways within fast-growing organisms better suited to the rigours of industry.

*Escherichia* *coli* is the well-established workhorse of the biotechnology world, so it should come as no surprise that basidiomycete NPs have been produced within this organism. The appeal is obvious, it is a unicellular, well understood and very fast-growing organism, and robust techniques and tools for its manipulation are available. Examples of its use include, the heterologous expression of sesquiterpene synthases from *Coniophora puteana* for use in generating β-copaene and cubebol, which were then used to create a heterologous microbial production system, with yields that exceeded all previously published results for these compounds (Mischko et al., 2018). The unspecific peroxygenase MroUPO from the basidiomycete *Marasmius rotula* was expressed in *E. coli* alongside computationally guided mutated variants for the purposes of optimising the enzyme (Carro et al., 2019). Cytochrome P450s from the basidiomycete *Phanerochaete chrysosporium* were expressed using the T7 RNA polymerase/promoter system in *E. coli* and subsequently optimised by modifying their N-terminal amino acid sequences, and their mechanisms of function elucidated (Hatakeyama et al., 2016). *Escherichia* *coli* was used to functionally characterise, through heterologous expression, putative sesquiterpene synthases from *Omphalotus olearius* (Wawrzyn et al., 2013). However, for expression of more complex proteins and entire biosynthetic pathways, *E. coli* is not appropriate, not just because the genes are not codon optimised for this organism, or because the systems for required post-translational modifications are not usually present, but because secondary metabolite excretion is not developed and because many of the useful NPs produced by basidiomycete fungi carry antibiotic properties, to which *E. coli* is susceptible.

Because of the limitations posed by *E. coli*, it is natural for researchers to look for a fungal substitute that retains the benefits of being a well-understood, generally recognised as safe to work with, fast growing, unicellular, model organism, with a plethora of cost efficient tools and techniques for transformation such as a golden gate toolkit (Lee et al., 2015). Yeasts fulfil all these roles and have a long history of industrial application making them simple to culture in bioreactors. Among yeasts, *Saccharomyces cerevisiae* has been used to study enzymes from basidiomycete fungi. For instance, three sesquiterpene synthase genes from the medicinal mushroom *Lignosus rhinocerotis* were identified using genome mining and transcriptomics, and were heterologously expressed in *S. cerevisiae* to give sesquiterpene alcohols (Yap et al., 2017). Many *S. cerevisiae* strains exist today which overproduce the precursor compounds utilised for biosynthesis of secondary metabolites, for example *S. cerevisiae* EPY300 overexpresses the mevalonate pathway, and can be exploited to produce terpenoids in large amounts (Ro et al., 2006). The more recently developed *S. cerevisiae* EPYFA3, which is derived from EPY300, overexpresses both the mevalonate and the shikimate pathways and is shown to be able to overproduce isoprenoid quinones (Kaur et al., 2020). *Pichia pastoris* is another popular yeast to work with, and the minor laccase isozymes Lac2 and Lac3 from the basidiomycete *Grifola frondosa* were heterologously expressed and characterised using this yeast (Nitheranont et al., 2017). *Pichia* *pastoris* was also used to heterologously express a further nine laccases from *Cerrena* sp. (Yang et al., 2015, 2016). Yeasts are also a good choice for heterologous expression because directed evolution can be used to rapidly optimise expression of a secondary metabolite from heterologous hosts. Compared to bacteria, which can also be used to perform directed evolution, yeasts are more likely to correctly express and secrete basidiomycete proteins, therefore enabling for in-host screening of the effects of directed evolution. For example, the unspecific peroxygenase from *Agrocybe aegerita* was engineered through directed evolution in *S. cerevisiae* to improve its catalytic attributes, and the resulting evolved unspecific peroxygenase was then inserted into *P. pastoris* to obtain a 27-fold increase in production levels (Molina-Espeja et al., 2015).

Despite some successful examples of using *S. cerevisiae* to express basidiomycete genes, this host is not always able to correctly express entire basidiomycete gene clusters. A recent study showed that only 7/17 (41%) basidiomycete gene clusters were productive when expressed in *S. cerevisiae*, as opposed to 17/26 (65%) ascomycete clusters (Harvey et al., 2018). Potential reasons may include the inability of *S. cerevisiae* to process the numerous introns found in basidiomycete genes. Indeed, fungi of the phylum Basidiomycota are known to be generally richer in introns than other fungi, with basidiomycete fungi showing 5.28 exons per gene while ascomycete fungi have only 2.58. Among fungi of the phylum Ascomycota, *S. cerevisiae* has only 1.04 exons per gene (Goffeau et al., 1996), while *A. oryzae* has 2.98 (Arnaud et al., 2011). Indeed, a recent study led by Oikawa's group showed that *A. oryzae* is able to remove 87% of the introns from basidiomycete terpene synthase genes (Nagamine et al., 2019). The same group used this host to study the biosynthesis of the diterpene erinacine, where 90% of introns were correctly spliced out by *A. oryzae* and the remaining introns were removed by PCR prior to achieving correct expression (Liu et al., 2019). *Aspergillus* *oryzae* has been successfully used in a number of studies to recreate and characterise basidiomycete NP pathways, such as those for pleuromutilin (Alberti et al., 2017a; Bailey et al., 2016; Yamane et al., 2017) and strobilurin (Nofiani et al., 2018), in which introns were removed prior to heterologous expression.

Among fungi of the phylum Basidiomycota, the corn smut *U. maydis* has recently been developed into a heterologous host for the production of terpenoids through the overexpression of endogenous mevalonate pathway genes (Lee et al., 2020). The chassis was validated through the heterologous expression of fungal and plant sesquiterpenoid synthases from *C. cinerea* and the Nootka cypress *Callitropsis nootkatensis*, respectively.

Often the best results for functional characterisation can be obtained by combining multiple heterologous systems into one study such as when six sesquiterpene synthases from *C. cinerea* were expressed in *E. coli* and *S. cerevisiae* (Agger et al., 2009).

### Molecular network analysis

The successful characterisation of basidiomycete NPs and their biosynthesis not only requires molecular biology tools for genetic engineering and heterologous expression, but also relies upon metabolic analyses (van der Hooft et al., 2020). Traditional bioactivity-guided fractionation of metabolite extracts has now been overtaken by more sensitive approaches, such as untargeted metabolomics and data analysis of tandem mass-spectrometry (MS/MS). MS/MS spectra result from the fragmentation of metabolites and constitute the fingerprint of a given compound. Once metabolite extracts have been generated and analysed through MS/MS, one can compare the data with spectral libraries. However, these libraries are still far from comprehensive. For instance, the Global Natural Product Social molecular networking (GNPS) site contains MS/MS reference spectra for only 2.5% of known NPs (van der Hooft et al., 2020). A parallel approach that can aid data dereplication and identification of unknown metabolites, as well as to link pathway intermediates, shunt and final products to each other, relies on the use of software that can group metabolites based on similarities in their MS/MS fragmentation patterns. GNPS (Wang et al., 2016) has been used by many NP researchers for this purpose, including for fungi of the phylum Basidiomycota. For instance, molecular network analysis enabled the discovery of two bioactive xylosides from co-cultures of the basidiomycete fungi *Trametes versicolor* and *Ganoderma applanatum* (Yao et al., 2016). In this study, a useful feature of molecular network analysis was highlighted when sub-structure predictions were made based on the grouping of metabolites that differ in mass by values equivalent to pentose moieties, suggesting that glycosylation would take place. A follow-up study by the same group investigated the origin of the metabolites that were induced by co-cultures of the two fungi to link them to the respective producing organism (Xu et al., 2018). The authors combined ^13^C-based labelling and molecular network analyses to show that 31 out of 74 induced features were being produced by either *T. versicolor* or *G. applanatum*, or by both fungi. Importantly, the origin of the induced metabolite could be discerned even when the structure of the molecule in question was unknown. This approach could be applied to other co-culture studies and aid the identification of cryptic NP gene clusters.

New features are frequently being added to the GNPS suite, such as the MS2LDA tool, which identifies sub-structures within a metabolite structure through the unsupervised detection of co-occurring molecular fragments, inspired by text-mining (van der Hooft et al., 2016). This tool enables users to annotate molecules for which no reference spectra can be found and helps reveal biochemical relationships between compounds. Another newly added feature to the GNPS platform is the Mass Spectrometry Search Tool (MASST), which allows researchers to perform MS searches (Wang et al., 2020b). With this tool, all public data available in GNPS can be searched using an MS/MS spectrum as a query, in a similar manner to searches using nucleotide and amino acid sequences through the popular Basic Local Alignment Search Tool (BLAST) (Altschul et al., 1990).

## Future prospects

In the last five years, recently developed technologies for genome sequencing and genetic engineering have been applied to basidiomycete fungi, including nanopore sequencing for the generation of long-read genome assemblies and CRISPR/Cas9-based editing. Future prospects will likely include the improvement of genetic engineering tools, for instance through the wider implementation of HDR via a repair template in the CRISPR/Cas9 basidiomycete toolkits. HDR promotes a more precise gene editing compared to NHEJ, with the latter causing insertions or deletions of various length, which can lead to a shift of the open reading frame. However, HDR is generally less efficient than NHEJ, and the inclusion of repair arms does not guarantee that it will take place. Ways of improving the efficiency of HDR in basidiomycete CRISPR/Cas9 endeavours may include the inhibition of enzymes involved in the NHEJ pathway. This approach was used to increase the efficiency of HDR genome editing in mice and mammalian cell lines by up to 19-fold by targeting DNA ligase IV using the inhibitor Scr7 (Maruyama et al., 2015). An alternative to canonical double-stranded break-mediated CRISPR/Cas9 editing can be found in base editing (Komor et al., 2016). This involves the use of a catalytically dead Cas9 and a cytidine or adenosine deaminase, which enable for base-editing without needing to cause double-stranded breaks in the target or to introduce a repair template. Successful CRISPR/Cas9-mediated base editing has recently been reported in filamentous ascomycetes (Huang et al., 2019). Techniques such as these will continue to be developed and will eventually cross over into basidiomycetes.

Besides the engineering of a NP-producing basidiomycete strain, heterologous expression of a candidate NP gene cluster is an established and effective approach to link specialised metabolites to their genetic determinants, discover new bioactive molecules and characterise their biosynthesis (Alberti et al., 2017b). The successful heterologous expression of basidiomycete clusters has been described in several hosts, including *S. cerevisiae* (Harvey et al., 2018) and, despite the reported incomplete intron-splicing, in *A. oryzae* (Nagamine et al., 2019). Ascomycete hosts are easier to transform and engineer than basidiomycete fungi, therefore they are the preferred chassis for the study of basidiomycete NP gene clusters. However, a first step toward the use of *U. maydis* has been achieved recently when its mevalonate pathway was overexpressed, and the resulting strain was used to express fungal and plant sesquiterpenoid synthases (Lee et al., 2020). Expression of entire biosynthetic gene clusters will likely follow in this host. Other model basidiomycete fungi that may be developed into heterologous hosts for the study of NPs include *C. cinerea* and *A. bisporus*, which, unlike *U. maydis*, benefit from not being plant pathogenic. Molecular toolkits (Burns et al., 2005; Collins et al., 2010) have been established for these two mushroom-forming species, followed by the development of CRISPR/Cas9-based engineering tools (Sugano et al., 2017; Waltz, 2016), and will likely be expanded and employed in the near future to aid the study of NPs made by other fungi of the phylum Basidiomycota. The establishment of a basidiomycete heterologous host means that genes of interest could be amplified from the genomic DNA of donor basidiomycete strain, or even be obtained as synthetic DNA, without having to embark in the laborious task of predicting and removing their numerous introns.

On the front of metabolic analyses, publicly available NP databases (e.g. The Natural Product Atlas; van Santen et al., 2019) and molecular network analysis (e.g. GNPS; Wang et al., 2016) represent valuable tools for data dereplication and NP characterisation. The in-silico prediction of NP structures based on gene sequences is a task that still requires refinement. Tools such as antiSMASH (Blin et al., 2019) and PRISM (Skinnider et al., 2017) can predict the sequence of monomers that are assembled into PKS and NRPS biosynthetic lines based on module specificity. However, the accurate prediction of a cyclised NP structure, as well as of the functionalisation of the core NP through tailoring reactions, still remain challenging. Future efforts are envisaged to take place that will aim to improve such predictions by training machine learning models to link genetic and metabolic data.

## Concluding remarks

Fungi belonging to the phylum Basidiomycota have shown the potential to produce bioactive metabolites that have been exploited commercially, including as antimicrobials (such as pleuromutilin antibiotics) and agrochemicals (such as strobilurin fungicides). Recent advances of molecular tools for the study of basidiomycete fungi are allowing researchers to explore this prolific microbial taxon further. Future endeavours will be necessary to improve the molecular toolbox available for the engineering of basidiomycete fungi. These, coupled with efficient and reliable transformation protocols, will likely lead to the development of a true basidiomycete heterologous host that can be employed to discover NPs from cryptic gene clusters and characterise their biosynthesis. Further efforts are also needed to improve the prediction of NP structures starting from genetic information, in order to navigate the vast number of whole-genome and metagenome sequences that are currently being generated and prioritise gene clusters for experimental characterisation.
